# Modifications on the Basic Skeletons of Vinblastine and Vincristine 

**DOI:** 10.3390/molecules17055893

**Published:** 2012-05-18

**Authors:** Péter Keglevich, László Hazai, György Kalaus, Csaba Szántay

**Affiliations:** Department of Organic Chemistry and Technology, University of Technology and Economics, H-1111 Budapest, Szt. Gellért tér 4, Hungary

**Keywords:** antitumor therapy, vinblastine, vincristine, derivatives

## Abstract

The synthetic investigation of biologically active natural compounds serves two main purposes: (*i*) the total synthesis of alkaloids and their analogues; (*ii*) modification of the structures for producing more selective, more effective, or less toxic derivatives. In the chemistry of dimeric *Vinca* alkaloids enormous efforts have been directed towards synthesizing new derivatives of the antitumor agents vinblastine and vincristine so as to obtain novel compounds with improved therapeutic properties.

## 1. Introduction

**Figure 1:**
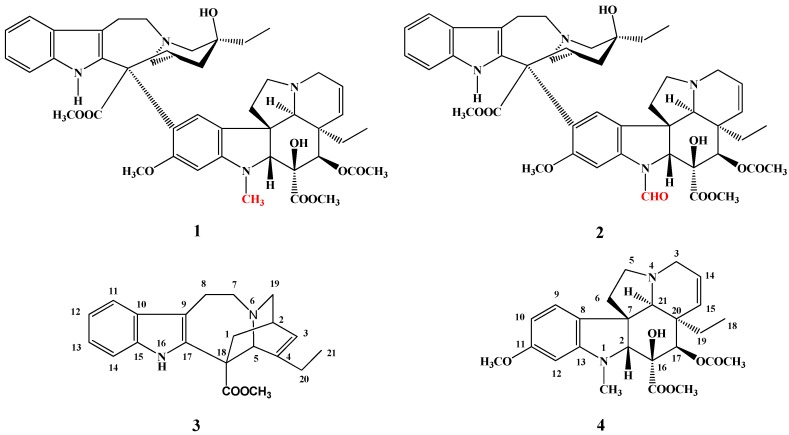
Structures of vinblastine (**1**) and vincristine (**2**).

Vinblastine (**1**) and vincristine (**2**) are dimeric alkaloids (Figure 1) isolated from the Madagaskar periwinkle plant (*Catharantus roseus*), exhibit significant cytotoxic activity and are used in the antitumor therapy as antineoplastic agents.

In the course of cell proliferation they act as inhibitors during the metaphase of the cell cycle and by binding to the microtubules inhibit the development of the mitotic spindle. In tumor cells these agents inhibit the DNA repair and the RNA synthesis mechanisms, blocking the DNA-dependent RNA polymerase.

Vinblastine (**1**) has two monomer alkaloid parts: catharanthine (**3**) and vindoline (**4**). The difference between vinblastine (**1**) and vincristine (**2**) is that the former has a methyl while the latter has a formyl group on the indole nitrogen of the vindoline skeleton (Figure 1).

The chemical and biological characteristics of these dimeric alkaloids are presented in several reviews, of which only two publications are mentioned here [1,2]. The aim of our present work, however, is to highlight the different derivatives and derivatisations of vinblastine (**1**) and vincristine (**2**), focusing primarily on selected cases that stand out from the enormous literature data owing to their interesting chemistry or important biological effects. 

## 2. Modification of Vinblastine

**Scheme 1:**
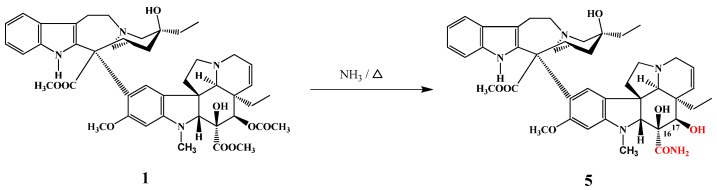
Preparation of 17-desacetylvinblastine-16-amide (**5**).

According to an Eli Lilly patent [3] the carboxylic ester group of vinblastine at position 16 was converted to amide **5** in a reaction with refluxing ammonia (Scheme 1).

Starting from the corresponding hydrazide, which was deacetylated at position 17, hydrogenolysis of the hydrazide resulted in the formation of 16-amide function [4]. 17-Desacetylvinblastine-16-amide (**5**), named VINDESINE, is an excellent antitumor agent, that can be used for the treatment of adenocarcinoma, lymphosarcoma and osteogen sarcinoma.

**Scheme 2:**
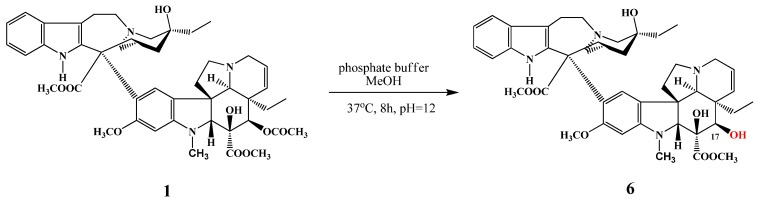
Selective desacetylation of vinblastine (**1**).

The 17-*O*-acetyl group of vinblastine was selectively hydrolysed by Thimmaiah and co-workers [5] using a phosphate buffer in methanol; thus 17-desacetylvinblastine (**6**) was successfully prepared in 95% yield (Scheme 2). Compound **6** can be considered as the active metabolite of vinblastine [6], because its activity is substantially higher than that of the prodrug vinblastine.

**Scheme 3:**
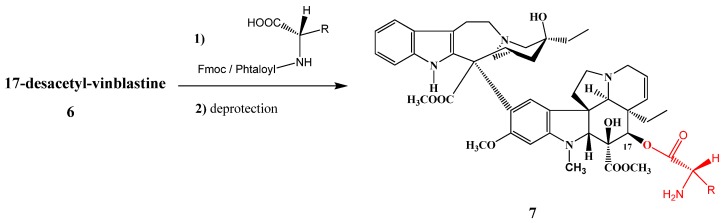
Amino acid derivatives of vinblastine.

The same reaction was carried out by Brady [6] in 95% yield with a 1:3 hydrazine-methanol mixture at 20 °C for 20 h. The resulting 17-desacetylvinblastine (**6**) was allowed to react with amino acids protected on the *N*-terminal with Fmoc or Phth [(9-fluorenylmethyloxi)carbonyl) and phthtaloyl]; after coupling the products were deprotected and new derivatives **7** containing amino acids (e.g., glycine, leucine and proline) and different peptides attached to position 17 with an ester bond were obtained (Scheme 3). Compounds **7** showed significant effects against prostate cancer.

**Scheme 4:**
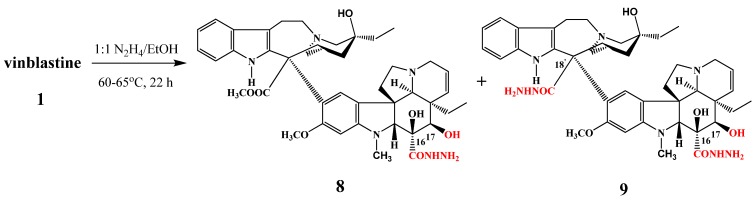
Hydrazide derivatives **8** and **9** of vinblastine (**1**).

Under treatment of vinblastine with a 1:1 mixture of hydrazine and ethanol at 60–65 °C for 22 h two products were obtained (Scheme 4): 17-deacetyl-16-hydrazidevinblastine (**8**) and 17-deacetyl-16-hydrazide-18′-hydrazidevinblastine (**9**).

**Scheme 5:**
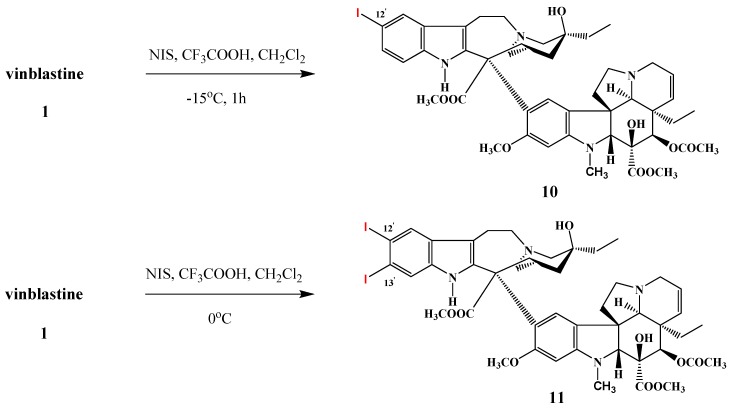
Iodination reactions on vinblastine (**1**).

Scott *et al*. synthesized vinblastine derivatives substituted at the 12′- and 13′-positions of the catharanthine monomer (Scheme 5). In reactions with *N*-iodosuccinimide in dichloromethane solution in the presence of trifluoroacetic acid at −15 °C for 1 h, 12′-iodovinblastine (**10**) was isolated in high yield. By performing the iodination reaction at 0 °C 12′,13′-diiodovinblastine (**11**) was obtained in 25% yield.

**Scheme 6:**
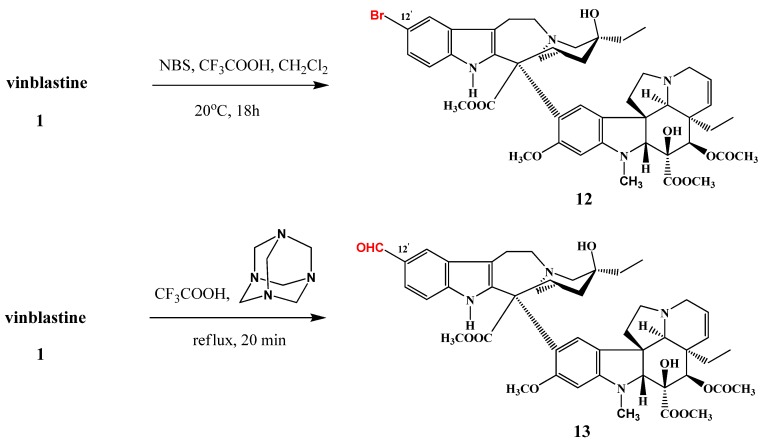
Bromination and formylation of vinblastine (**1**).

12′-Bromovinblastine (**12**) was prepared in 69% yield (Scheme 6) in the reaction of vinblastine (**1**) with *N*-bromosuccinimide. Reaction between hexamethylenetetramine and vinblastine under reflux for 20 min resulted in the corresponding formyl derivative (**13**) in 40% yield [7].

**Scheme 7:**
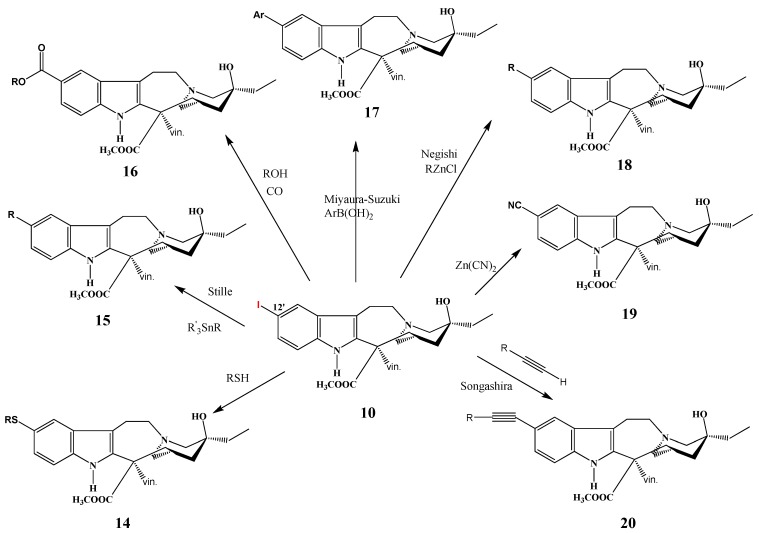
Reactions of 12′-iodovinblastine (**10**).

Numerous derivatives of vinblastine on the catharanthine part (compounds **14**–**20**, Scheme 7) were prepared by the same research group from 12′-iodovinblastine (**10**), primarily by coupling reactions catalyzed by palladium (e.g., Stille, Songashira, Negishi) [8]. SAR of derivatives were investigated on HeLa (cervical cancer) and MCF-7 (breast cancer) cell lines and several derivatives showed promising anticancer activity in the P388 murine leukemia model.

**Scheme 8:**
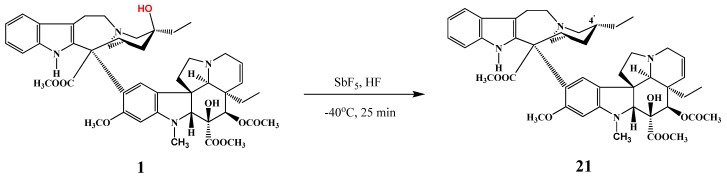
Reduction of vinblastine (**1**).

Selective removal of the 4′-hydroxy group of vinblastine was performed by Lafitte starting from vinblastine (**1**). In the reaction with antimony(V) pentafluoride and hydrogen fluoride at −40 °C for 25 min (Scheme 8) 4′-desoxyvinblastine (**21**) was obtained in 63% yield [9]. The same procedure resulted in the derivatives of anhydrovinblastine (**32**) and vinorelbine (**33**) with a reduced C=C double bond.

**Scheme 9:**
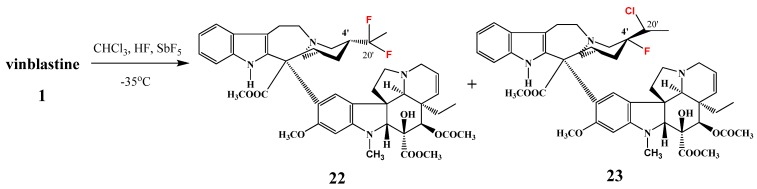
Fluorination reaction of vinblastine (**1**).

Fahy and co-workers also treated vinblastine (**1**) with antimony pentafluoride and hydrogen fluoride in chloroform at −35 °C and two new derivatives of vinblastine were isolated (Scheme 9): 4′-deoxy-20′,20′-difluorovinblastine (**22**) in 50% yield and 4′-deoxy-4′-fluoro-20′-chlorovinblastine (**23**) in 6% yield [10]. 

**Scheme 10:**
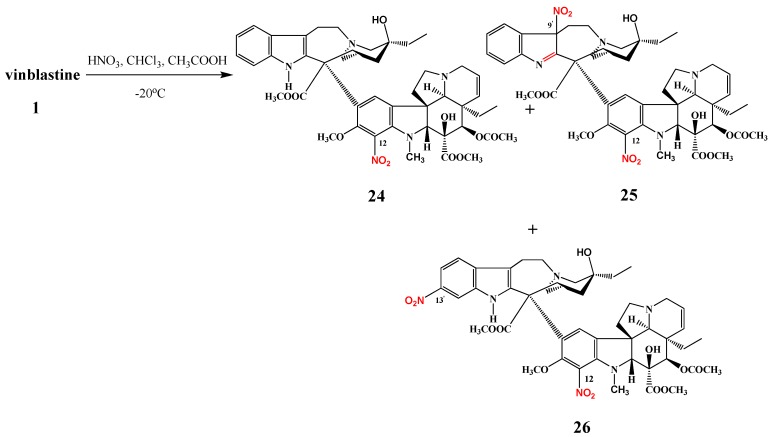
Nitration of vinblastine (**1**).

The nitration of vinblastine (**1**) was investigated by Szántay *et al*. [11]. Vinblastine (**1**) was allowed to react with nitric acid in an acetic acid/chloroform solution at −20 °C and three products were isolated (Scheme 10): 12-nitrovinblastine (**24**) in 49% yield, a 9′,12-dinitrovinblastine derivative **25** in 9% yield, and 12,13′-dinitrovinblastine (**26**) in 6% yield [12]. After reduction of the corresponding nitro derivatives some of the aminovinblastines obtained had important antitumor activity in non-small cell lung cancer, in breast and colon cancer and in leukemia.

Vincristine (**2**) was also nitrated under similar conditions, and in this case the nitro group attacked the 11′-, 9′- or the 13′-positions. All of the derivatives, amongst which 13′-nitrovincristine (**40**) was the major product, were isolated [13].

**Scheme 11:**
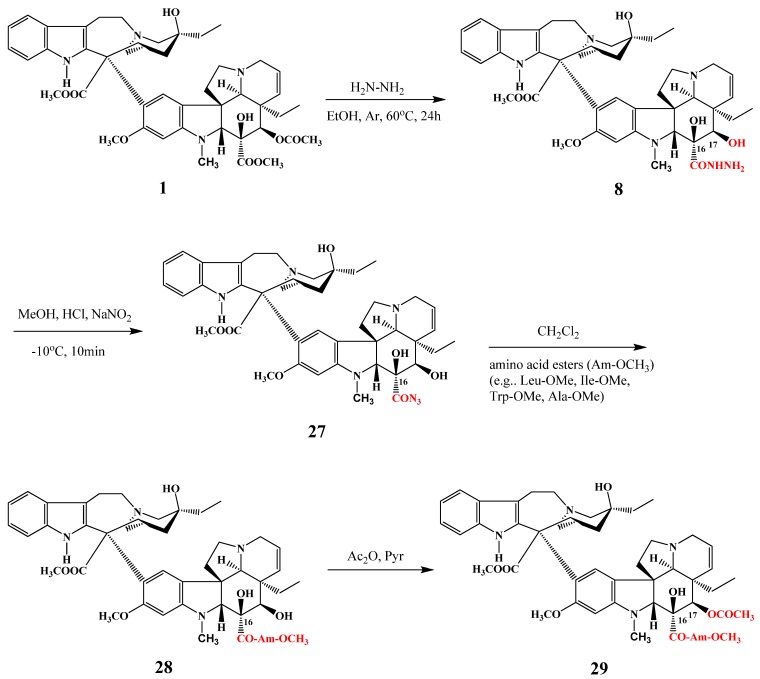
Coupling of vinblastine (**1**) with amino acids.

Rao and co-workers synthesized 17-desacetylvinblastine-16-hydrazide (**8**) from vinblastine (**1**), then the azide **27** was prepared and without isolation of the intermediate azide, vinblastine (**1**) was coupled with different amino acids through their amino group giving compounds **28** (Scheme 11). Finally, in most cases, the 17-hydroxy substituent was acetylated again to give **29** [14]. Most of the new compounds showed cytotoxic activity against P388 and L1210 leukemia, melanoma, breast cancer and small cell lung cancer [15].

**Scheme 12:**
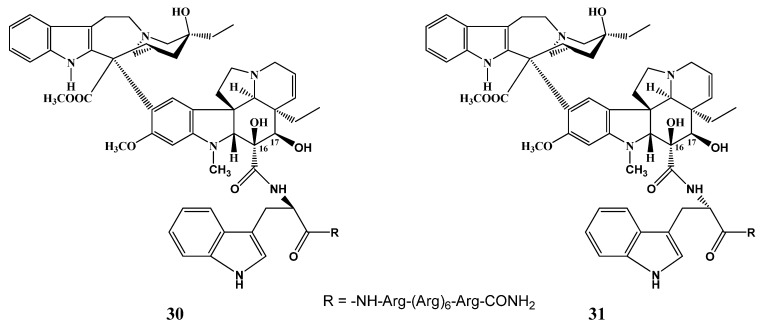
Vinblastine derivatives conjugated with carrier peptide.

D- and L-Tryptophan derivatives of the 16-position of desacetylvinblastine were conjugated through the carboxyl group with oligoarginine octapeptide as a carrier peptide at the *N*-terminus (compounds **30**,**31**) by Bánóczi *et al*. [16] (Scheme 12). One of the stereoisomers **30** or **31** showed a selective cytotoxic effect against the HL-60 human leukemia cells of higher proliferation rate.

**Scheme 13:**
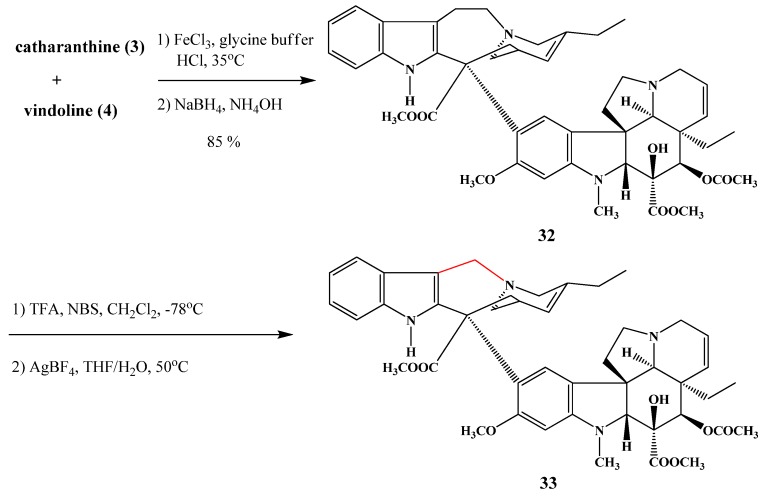
Synthesis of vinorelbine (**33**).

Ngo *et al.* [17] synthesized vinorelbine (**33**) in two steps from anhydrovinblastine (**32**), prepared by the coupling of vindoline (**4**) and catharanthine (**3**) (Scheme 13). This derivative is a potent antitumor agent in the treatment of non-small cell lung cancer [18]. In addition vinorelbine (**33**) and anhydrovinblastine (**32**) were hybridized through the 17-hydroxy group of the vindoline part with colchicine, podophyllotoxine and baccatine III to investigate the effect of the new molecules on the polymerisation of tubuline [19].

Ngo and his research group hybridized the cleavamine moiety of anhydrovinblastine (**32**) and vinorelbine (**33**) on the tertiary amine part with the antimitotic cyclopeptide phomopsin-A and obtained some potent inhibitors of the microtubules assembly and derivatives of good cytotoxicity against KB human cell lines [17]. Vinblastine (**1**) was also conjugated with a folic acid unit by the azide coupling method presented by Vlahov *et al*. [20].

**Scheme 14:**
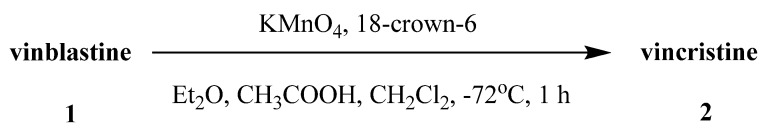
Oxidation of vinblastine (**1**) to vincristine (**2**).

A number of vinblastine congeners were synthesized by Kuehne and co-workers, first of all by changing the piperidine ring of catharanthine (**3**), which were potent against leukemia and colon cancer cell lines. Vinblastine (**1**) was successfully oxidized to vincristine (**2**) (Scheme 14) by the same research group, using potassium permanganate and 18-crown-6 phase transfer catalyst [21].

**Scheme 15:**
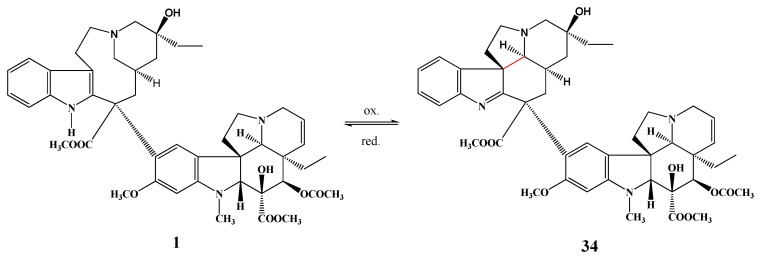
Ring transformation reaction.

Szántay *et al*. prepared cyclovinblastine (**34**) by oxidation of vinblastine (**1**). The ring transformation reaction of cyclovinblastine (Scheme 15) and cyclovincristine was also investigated and the structure of the products was identified [22].

**Scheme 16:**
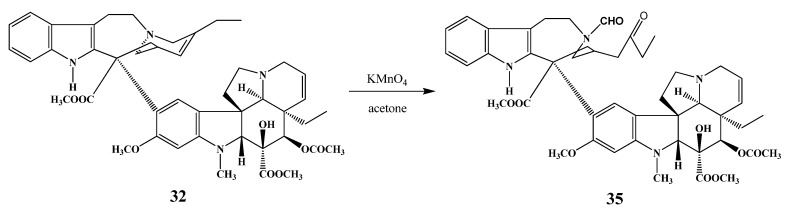
Preparation of vinamidine (**35**).

Kutney *et al*. prepared vinamidine (**35**) by the reaction of anhydrovinblastine (**32**) with potassium permanganate (Scheme 16) in acetone solution [23].

Bornmann and Kuehne elaborated the total synthesis of vinamidine (**35**) and some similar alkaloids [24]. In the course of the reaction procedure a new tetracyclic key intermediate was synthesized, which could be used for preparation of other similar alkaloids.

## 3. Changes in the Structure of Vincristine

**Scheme 17:**
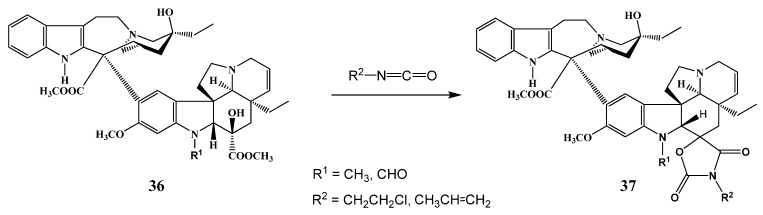
Spiro-substituted dimer alkaloids.

Spiro-oxazolidinedione substituted derivatives (Scheme 17) of vincristine [and similarly of vinblastine (**37**)] were synthesized starting from the corresponding 17-deacetoxy basic skeletons [25]. The key step of the reaction procedure was the reaction of the dimers **36** with isocyanates. In the course of the synthesis compounds were obtained with excellent cytotoxic activity and the new derivatives were active against leukemia.

**Scheme 18:**
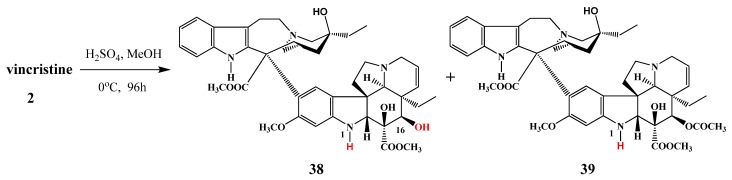
Deacetylation of vincristine (**2**).

Thimmaiah *et al*. treated vincristine (**2**) with sulfuric acid in methanol (Scheme 18) and 17-deacetyl-*N*-desformylvincristine (60%) (**38**) and *N*-deformylvincristine (40%) (**39**) were isolated [5].

**Scheme 19:**
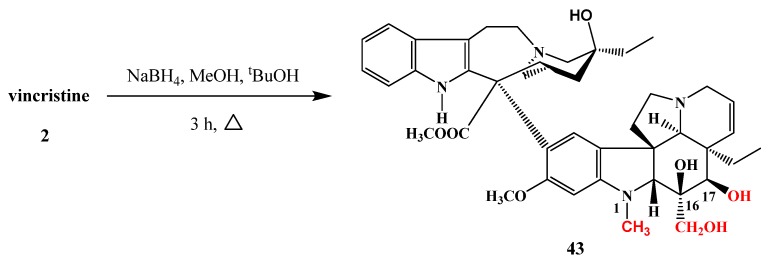
Reduction of vincristine (**2**).

The reduced derivative of vincristine (**43**) was prepared by Szántay and his co-workers (Scheme 19). The reduction was carried out with sodium borohydride in a mixture of alcohols in 63% yield [26].

**Scheme 20:**
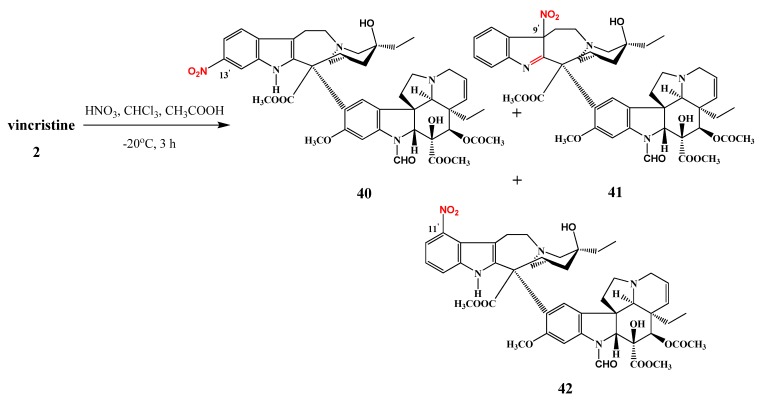
Nitration of vincristine (**2**).

The same research group investigated the nitration reaction also of vincristine (**2**) and three of the products were isolated (Scheme 20): 13′-nitrovincristine (**40**) as a major products (60%), a derivative of 9′-nitrovincristine (**41**) (31%) and 11′-nitrovincristine (**42**) in 5% yield [12].

**Scheme 21:**
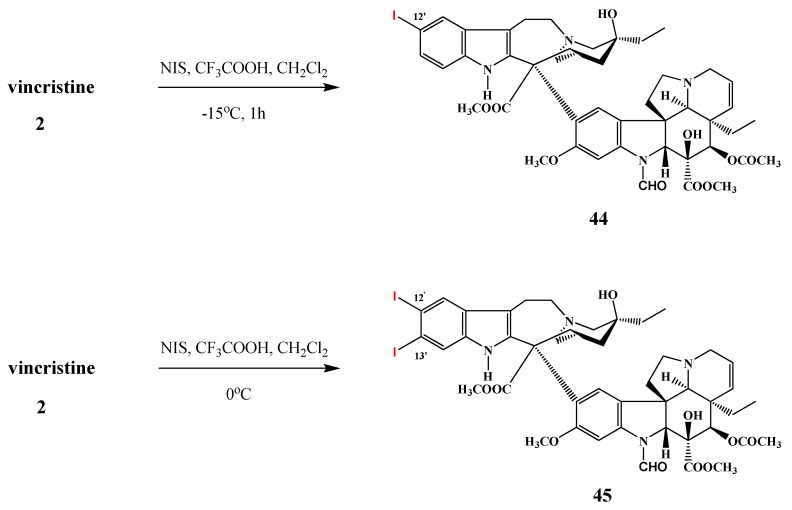
Iodinated derivatives of vincristine (**2**).

Scott *et al.* synthesized vincristine derivatives substituted in the catharanthine part at the aromatic ring in positions of 12′ and of 13′ (Scheme 21). 

**Scheme 22:**
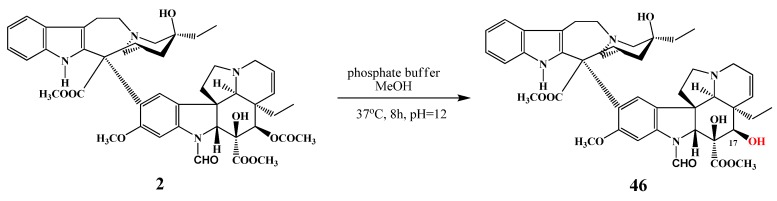
Selective deacetylation of vincristine (**2**).

The reaction between vincristine (**2**) and *N*-iodosuccinimide in dichloromethane in the presence of trifluoroacetic acid at −15 °C for 1 h resulted in 12′-iodovincristine (**44**) in 91% yield. When this reaction was carried out at 0 °C, 12′,13′-diiodovincristine (**45**) was obtained in 18% yield [7]. Thimmaiah *et al*., similarly to vinblastine (**1**), succeded in realizing the selective hydrolysis of vincristine (**2**) in the position of 17 (Scheme 22). Thus, 17-deacetylvincristine (**46**) was prepared in the reaction with phosphate buffer in methanol at pH 12 in 95% yield [5]. Cyclovinblastine (**34**) was successfully oxidized to cyclovincristine by Szántay with cromyl acetate [27]. 

Ahn *et al*. prepared *N*-formylcatharinine with enzymatic methods. This compound differs from vinamidine in position 1 of the vindoline part containing a formyl group instead of a methyl substituent [28].

**Scheme 23:**
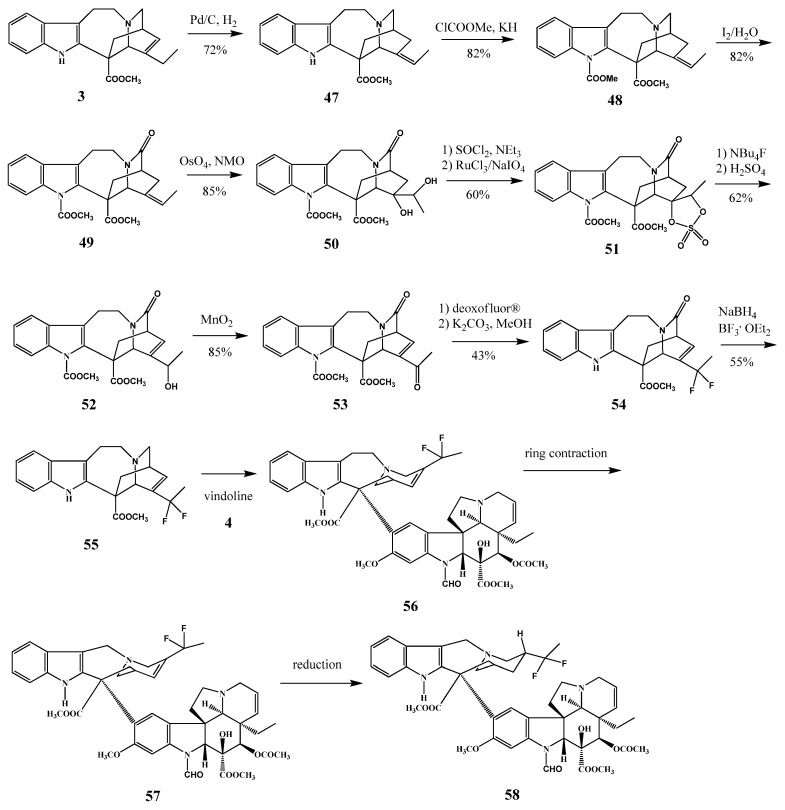
Detailed synthesis of vinflunine (**58**).

## 4. Modifications on the Catharanthine Skeleton

Moisan and his co-workers synthesized 20,20-difluorocatharanthine (**55**) starting from catharanthine (**3**) (Scheme 23). The latter difluoro derivative, when coupled with vindoline resulted in vinflunine (*Javlor*) (**58**) which is a vinorelbine (**33**) derivative [29]. The pharmacology of this new fluorinated *Vinca* alkaloid was fully investigated [30] and Phase III results are also presented [31].

Superacids were used in some cases to carry out the fluorination reactions [32], and the mechanism of the superacidic fluorination was investigated in detail [33]. The preparation of the vinflunine (**58**) was also elaborated directly by fluorination of vinorelbine (**33**) or by fluorination of anhydrovinblastine (**32**) and then with C′-ring contraction [34].

**Scheme 24:**
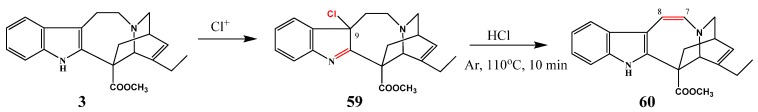
Preparation and reaction of 9-chlorocatharanthine (**59**).

Andriamialisoa *et al*. successfully prepared 9-chlorocatharanthine (**59**), and starting from this compound Δ^7^-catharanthine (**60**) was obtained (Scheme 24) [35]. Further reactions resulted in 7′-nor derivatives.

**Scheme 25:**
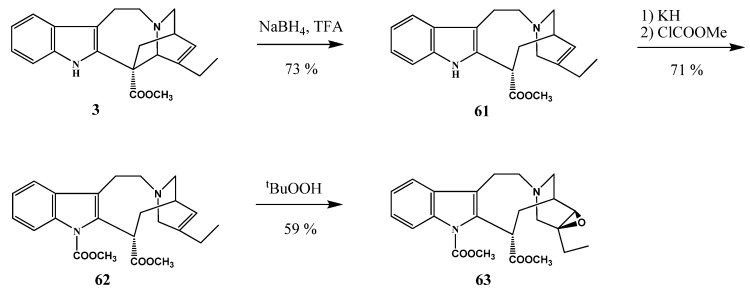
Building in an oxirane ring to catharanthine (**3**).

Catharanthine **63** containing an oxirane ring (Scheme 25) was prepared in the reaction of compound **62** with *tert*-butyl peroxide by Hardouin and his co-workers [36] who used this intermediate in the synthesis of leurosine. The latter was converted to anhydrovinblastine (**32**) which can be considered as the key intermediate for the synthesis of vinorelbine (**33**).

**Scheme 26:**
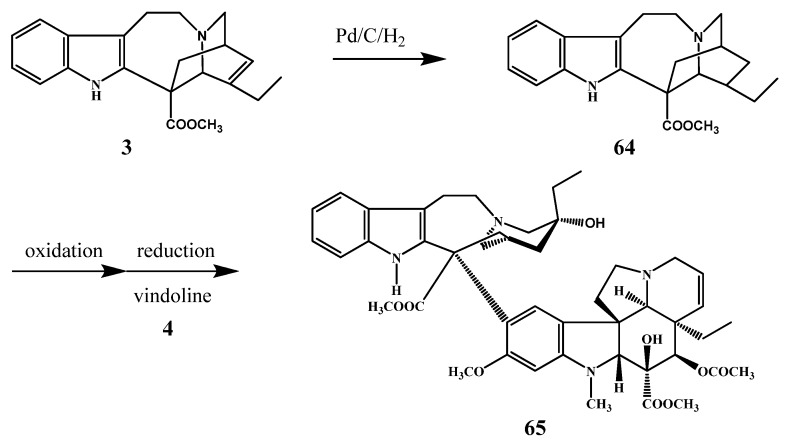
Preparation of leurosidine (**65**).

Catharanthine (**3**) was at first hydrogenated catalytically by Potier (Scheme 26) obtaining the saturated derivative **63** and after coupling with vindoline (**4**), leurosidine (**65**) was obtained [37]. Tam *et al.* prepared a number of derivatives of catharanthine and then these new derivatives were coupled with vindoline (**4**) (*see* Section 6. Coupling reactions) [38].

**Scheme 27:**
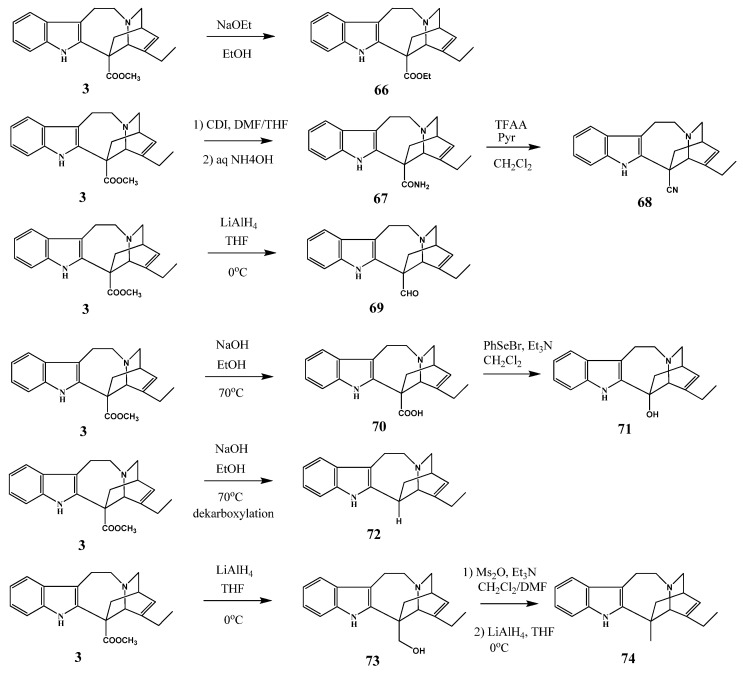
New derivatives of catharanthine.

In the course of the mentioned research project the ester group in position 18 of catharanthine was converted to other acid derivatives [38], e.g., amide, nitrile, and to different substituents such as aldehyde, hydroxy, alkyl, *etc*. by standard methods (Scheme 27). The aim of this work was to obtain new dimer alkaloids substituted in the catharanthine monomer.

**Scheme 28:**
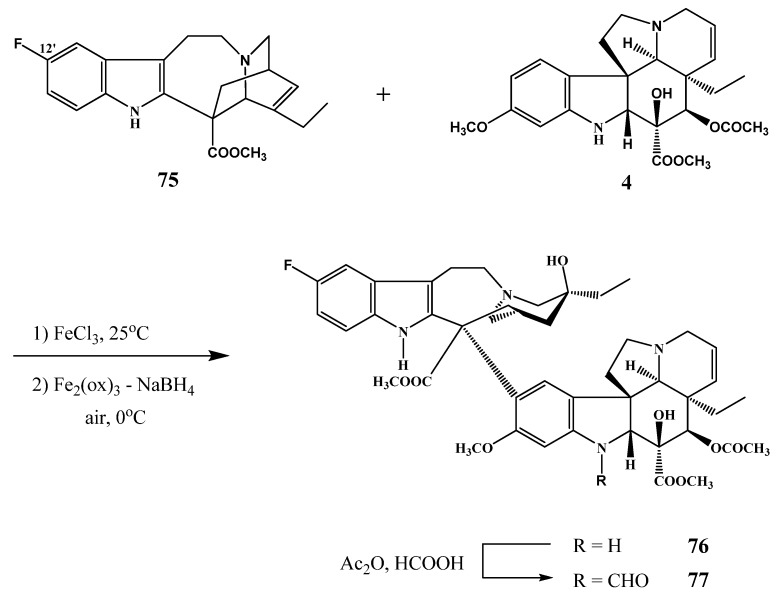
Fluoro-substituted dimeric alkaloids.

Numerous derivatives of vinblastine and vincristine were prepared by Boger [39]. These compounds were synthesized by the coupling of catharanthine substituted at the aromatic ring in position 12′ and vindoline (**4**) (Scheme 28). The new derivatives contained different substituents (nitro, amino, halogene, nitrile, alkyl, alkoxy, and thioalkyl), but compounds **76** and **77** proved to be the best antitumor molecules in the case of both sensitive and resistent human colon cancer cells.

## 5. Derivatizations of Vindoline

According to the literature the monomer *Vinca* alkaloid vindoline (**4**) was generally presumed and found to be ineffective against cell proliferations. Reactions and derivatives of vindoline for the synthesis of vinblastine were scarcely investigated. The reactivity of the aromatic ring of vindoline was presented by Szántay *et al*. [40], but the new vindoline derivatives were not used for the preparation of dimer alkaloids. 

**Scheme 29:**
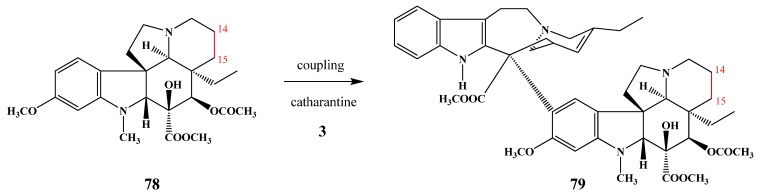
Coupling with saturated vindoline.

Kutney *et al.* [41] and Ishikawa [42] synthesized 14,15-dihydrovindoline (**78**), and its coupling with catharanthine resulted in 14,15-dihydroanhydrovinblastine (**79**) (Scheme 29). Meanwhile 14,15-dihydrovinblastine was simply prepared by catalytic hydrogenation of vinblastine, and its mechanism of action was different from that of the original vinblastine and the antitumor activity was rather low [43].

**Scheme 30:**
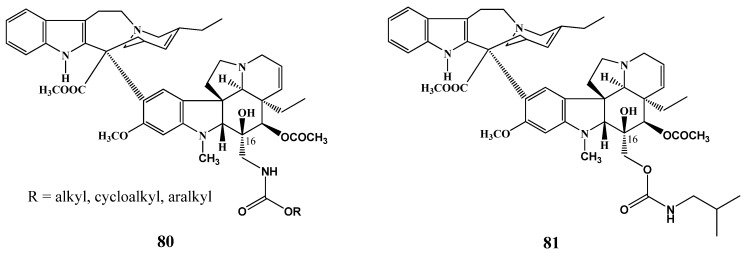
Carbamates of vinblastine.

One of the examples is the synthesis of carbamates of vindoline in position 16 (Scheme 30) and after coupling with catharanthine (**3**) new derivatives **80** and **81** of vinblastine were obtained which had important anticancer activity against human non-small cell lung cancer and on human cervix epithelial adenocarcinoma cell lines [44].

**Scheme 31:**
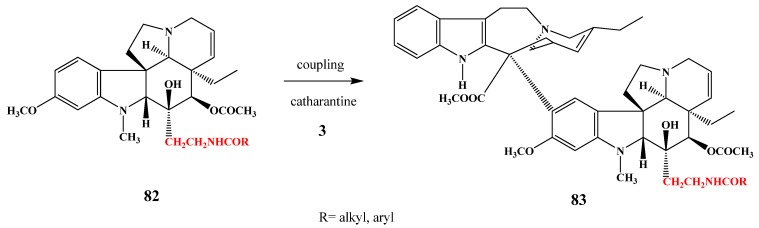
Amide substitued anhydrovinblastine derivatives.

Amide substituted anhydrovinblastines **83** were synthesized by building in acylated aminoethyl substituents in position 16 of the vindoline monomer **82** (Scheme 31). After coupling with catharanthine (**3**) new derivatives showed proliferation inhibition against HeLa cells [45].

**Scheme 32:**
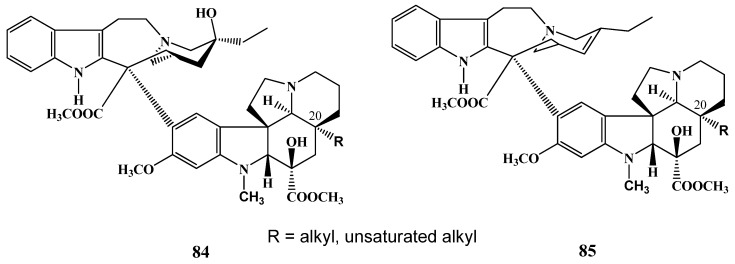
Modification of C-20 ethyl substitutent of vindoline.

The C-20 ethyl substituent of vindoline was changed to alkyl and alkene groups by another research group [46]. The new vindoline derivatives were coupled with catharanthine (**3**) resulting in new C-20 alkyl and alkenyl vinblastines **84** and **85** (Scheme 32). Biological investigations on colon cell lines were presented discussing the role of the absence or the presence of the C-20 ethyl group on the surrounding tubulin binding site.

**Scheme 33:**
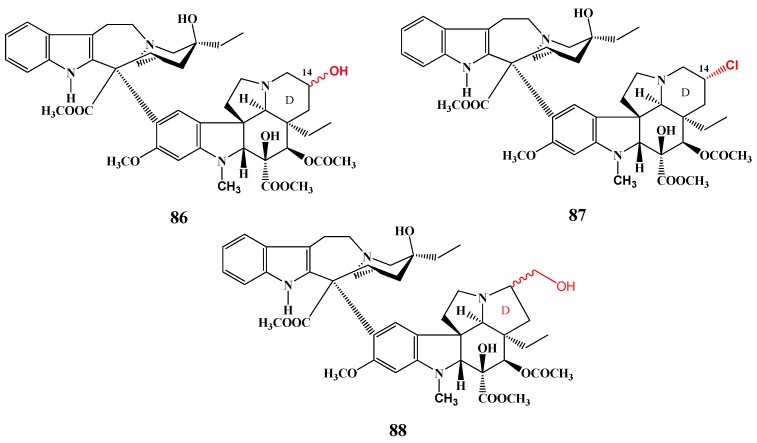
Vinblastines substituted at the vindoline D-ring.

Sasaki and his co-workers synthesized new derivatives of vindoline substituted in the D-ring (Scheme 33). After coupling by the usual way with catharanthine (**3**), new vinblastine derivatives were obtained containing a hydroxy group (compound **86**) or chloro atom (compound **87**) in the vindoline moiety. Moreover, a new derivative **88** was also prepared containing a five membered D-ring in place of the six-membered ring in the vindoline part of the dimer [47].

## 6. Coupling Reactions

**Scheme 34:**
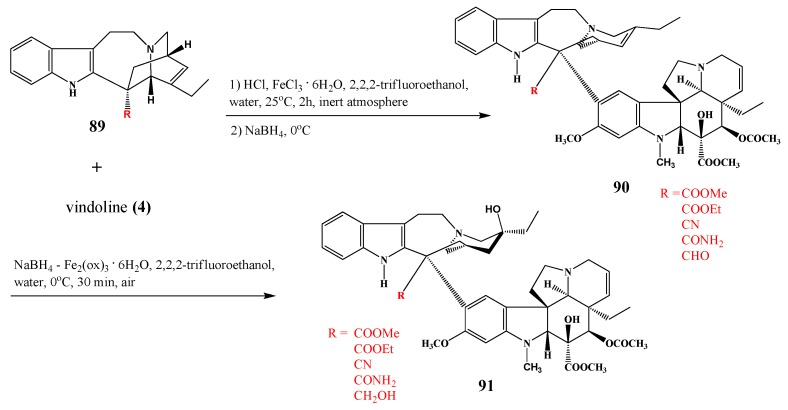
Coupling vindoline (**4**) with substituted catharanthines.

In this section only some tipical example resulting in new derivatives of dimeric alkaloids are mentioned from the many coupling reactions presented in the literature. Tam *et al*. [38] successfully coupled in two reaction steps the vindoline part with catharanthine substituted in the position 18′ shown before (*cf*. Scheme 27). The anhydro intermediates **90** were then oxidized to the expected target molecules, which are vinblastines **91** substituted in the catharanthine monomer (Scheme 34). 

**Scheme 35:**
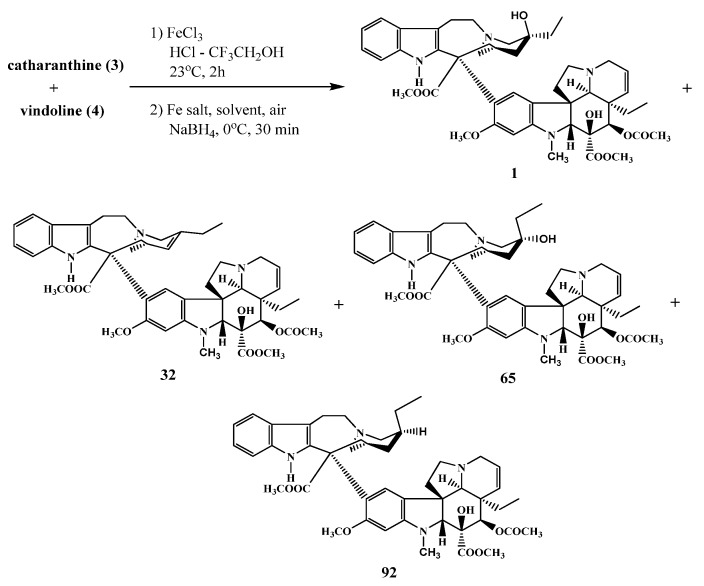
Coupling in a one-step reaction.

Ishikawa and his research group [42] elaborated the preparation of vinblastine by coupling catharanthine and vindoline in one step (Scheme 35). Depending the kind of Fe salt and the solvents used, products [vinblastine (**1**), anhydrovinblastine (**32**), leurosidine (**65**) and desoxyleurosidine (**92**), respectively] and their yields could be changed 

## 7. Conclusions

Over the years a number of research teams have performed extensive and valuable work to synthesize new derivatives of vinblastine and vincristine. Modifications in the vindoline skeleton or in the catharanthine moiety resulted in a number of new antitumor agents with more selectivity or less toxic properties. The mechanism of activity of *Vinca* alkaloids was investigated using these new derivatives and some new important results were found in connection with the tubulin polymerisation system. Currently, the structure of these dimers still seems to be an inexhaustible source of further research in this field of chemistry and therapy. 
